# Hepatitis B Virus-Related Liver Disease and Gut Microbiota: An Updated Review

**DOI:** 10.3390/microorganisms13112445

**Published:** 2025-10-25

**Authors:** Duoduo Lv, Ning Han, Huan Liu, Hong Tang

**Affiliations:** 1 Center of Infectious Diseases, West China Hospital of Sichuan University, Chengdu 610041, China; lvduoduo@scu.edu.cn (D.L.); hn950917@foxmail.com (N.H.); liuhuanxcw@163.com (H.L.); 2 Laboratory of Infectious and Liver Diseases, Institution of Infectious Diseases, West China Hospital of Sichuan University, Chengdu 610041, China; 3 Division of Infectious Diseases, State Key Laboratory of Biotherapy, Sichuan University, Chengdu 610041, China

**Keywords:** hepatitis B virus, gut microbiota, cirrhosis, hepatocellular carcinoma, probiotic, fecal microbiota transplantation

## Abstract

Although gut microbiota plays a pivotal role in numerous biological functions (e.g., energy, nutrients, metabolism, and immunological processes), growing evidence demonstrates that the gut microbiota is involved in the progression of liver diseases. The liver can be greatly influenced by alterations in intestinal microbiota due to increased gut permeability, allowing for the entry of bacterial products into the liver through the gut–liver axis. Recently, clinical and experimental research findings have demonstrated that microbiota dysbiosis plays an important role in the pathogenesis and progression of HBV-related liver diseases. In this review, we provide an overview of the gut microbiota and the microbiota–gut–liver axis in health; review HBV infection interactions with microbiota; discuss the role of microbiota dysbiosis in the pathogenesis of HBV-related liver disease, such as chronic HBV infection, liver cirrhosis, and hepatocellular carcinoma; and, finally, assess the potential for microbiota-targeted therapies, such as probiotics and fecal microbiota transplantation. This review will provide novel insights into individualized therapy for CHB patients based on gut microbiota alteration.

## 1. Introduction

Hepatitis B virus (HBV) infection is a global public health problem that threatens human health, and it is estimated that more than 296 million people are chronically infected with the it worldwide [1]. Chronic HBV infection (CHB) can cause serious complications such as liver fibrosis and cirrhosis, liver failure, and even hepatocellular carcinoma (HCC) [2,3]. Current therapies using nucleoside analogs and interferons are effective in inhibiting HBV replication. However, antiviral drug treatment cannot remove all viruses in liver cells. Even though the application of the hepatitis B vaccine has been reducing the HBV infection rate every year [4], CHB is still a heavy economic burden and health threat to many families. Therefore, the search for better ways to control the disease has begun to attract serious thought.

Emerging evidence shows that there are strict interactions among intestinal microflora, health, and disease—that is, unfavorable changes in the composition of intestinal microflora will alter host–microbiota interaction and host immune system progression [5]. Of note, the connection between the gut and the liver, the gut–liver axis, has been investigated more deeply. Recently, a large number of studies have suggested that alterations in intestinal microbiota seem to play a pivotal role in induction and promotion of the progression of liver diseases. There is growing evidence demonstrating that dysbiosis of the microbiota is associated with liver diseases, including alcoholic disease and nonalcoholic fatty liver disease [6], primary sclerosing cholangitis, liver fibrosis, cirrhosis and HCC [7]. As for CHB, many studies report that the composition of its microbiota is different to that of healthy subjects. With the development of genomic research and high-throughput sequencing technology, more researchers are increasingly paying more attention to the role of the gut microbiota in the progression and treatment of CHB. However, the exact relationship between HBV-related disease and human intestinal microflora is still unclear.

Therefore, in this paper, we review the relationship between the gut microbiota and HBV infection, describe related clinical trials involved in the composition and structure of gut microbiota in HBV-related liver disease, elucidate the main roles of intestinal microbiota in the progression of CHB, and discuss the use of probiotics and fecal microbiota transplantation (FMT) in HBV-related disease.

## 2. Gut Microbiota and Gut–Liver Axis in Humans

**Figure 1:**
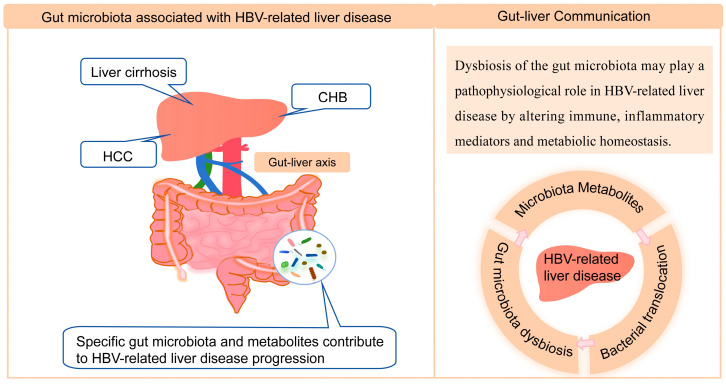
Schematic view of the link between gut microbiota and HBV-related liver disease. HBV-related liver disease by itself is associated with profound alterations in gut microbiota and damage at the different levels of defense of the intestinal barrier. In this setting of disruption of intestinal microbiota composition, concurrent damage to the epithelial and vascular intestinal barriers enables the passage of PAMPs and viable bacteria to the systemic circulation. The relevance of the severe disturbance of the intestinal barrier in HBV-related liver disease has been linked to translocation of live bacteria, bacterial infections, and disease progression.

The gut microbiota and humans have co-evolved, and various symbiotic interactions between the human host and the intestinal microbiota are necessary to maintain human health. The term “gut–liver axis” was created to highlight the close anatomical and functional relationship between the intestinal tract and the liver. The liver receives approximately 70% of its blood supply from the gut by the portal system. Most of the portal blood flow carries digestive products, but gastrointestinal pathogens or gut-derived antigens can also reach the liver via the gut–liver axis [8]. Multiple hepatic immune cells, including macrophages, lymphocytes, natural killer cells, and dendritic cells, respond to the continuous influx of these intestinal antigens or pathogens [9,10] and cause a unique local immune environment that regulates the immune tolerance of the liver [11]. Although there are many causes of liver damage (e.g., viruses, toxicity, and metabolism), the trigger mechanisms of various diseases (such as inflammation, steatosis, fibrosis, and cirrhosis) share commonalities, that is, gut microbiota dysbiosis is closely associated with many chronic liver diseases. Indeed, in the case of gut microbiota dysbiosis, damage to the integrity of the intestinal barrier and an excessive immune response of the liver to enterogenous factors and subsequent proinflammatory reactions potentially result in the occurrence and development of chronic liver disease [12,13]. An increasing number of studies have shown that there is a link between intestinal microbiota dysbiosis and disease progression in CHB patients through the gut–liver axis (Figure 1).

## 3. Gut Microbiota Modulate Immune Response During HBV Infection

### 3.1. Gut Microbiota and Host Immune Response

The intestinal flora plays a vital role in the development of the immune system. Early studies on germ-free (GF) mice demonstrated that the absence of commensal microbes is associated with impaired immune development, reduced intestinal lymphocyte numbers, and decreased levels of antimicrobial peptides and immunoglobulin (Ig)A [14]. However, rebuilding GF mice with gut microflora is sufficient to restore these immune system defects and abnormalities [15]. In fact, in GF mice, intestinal microbiota fundamentally guides the development of a normal intestinal physiology and functioning of the intestinal immune system, including various bacterial species enteroviruses [16,17]. Most recent reports have abundantly shown that the main role of microbiota in the immune system is to induce an immune response and enhance host–microbiota homeostasis. For example, the microbiota can promote the induction and/or activation of T-regulatory (Treg) cells at mucosal site [18]. The interleukin (IL)-10 produced by Treg cells, which has been proven to play a central role in regulation of intestinal homeostasis by inhibiting the immune response to microbial antigens [19]. Probiotics can induce the production of IL-10 Treg cells in vitro by regulating the function of dendritic cells [20]. Furthermore, systematic analysis of germ-free mice showed that the colonization of the entire mouse microbiota can coordinate a wide range of proinflammatory T helper (Th)1, Th17, and regulatory T cell responses [21].

Gut microbiota-released metabolites produced by commensal microbes may exert indispensable actions on host immunity. Furusawa et al. demonstrated that fatty acids (butyrate) obtained from intestinal microorganisms exert an epigenetic switch effect by increasing the histone H3 acetylation level of *Foxp3*, thereby promoting the differentiation of regulatory T cells [22]. Moreover, butyrate can drive monocyte-to-macrophage differentiation via histone deacetylase 3 inhibition, thereby amplifying antimicrobial host defense [23]. Also, there is ample evidence that short-chain fatty acids (SCFAs) produced by some of the anaerobic intestinal microbes can be considered key immune media [24]. In instance, Myung H Kim et al. demonstrated that SCFAs mediate the generation of the protective immune response in mice by promoting the rapid production of chemokines and cytokines [25]. Notably, SCFAs also promote the production of IL-10 in Th1, Th17, and Treg cells [26]. Collectively, these data pinpoint the components from the intestinal microbiota that play a vital role in inducing and promoting the host’s immune response.

### 3.2. Gut Microbiota Modulates the Immune Response Against HBV Infection

An innate and adaptive immune response is considered an important feature of the occurrence and development of HBV infection. Not only are a variety of immune cells and cytokines involved in the initiation and modulation of immune responses, but they can also activate the downstream antiviral signal pathways to directly or indirectly inhibit HBV replication [27,28]. For instance, hepatocellular recognition by HBV-specific effector CD8+ T cells leads to antiviral cytokine production and hepatocyte killing [29]. Clearly, the activation of the host’s anti-HBV immune response can effectively reduce the risk of HBV infection.

Chronic HBV infection is a complex and dynamic disease characterized by an un-predictable clinical course. One possible explanation is the developmental differences in immune function between infants and young children compared to adults [30]. Indeed, previous clinical studies have shown that the genetic structure of the host is a key factor in determining the outcomes of HBV infection [31], and the age of exposure to HBV is an important non-genetic factor in determining virus clearance [30,32]. It is noteworthy that the establishment of the intestinal microbiota plays a definitive role in the immune clearance of age-related HBV infection [33]. The adult gut microbiota has reached a higher steady state while the immune systems of children are immature and their microecological balance is unstable [34,35]. HBV may exploit the immature hepatic immune environment to establish chronic infection. Following maturation of the host immune system and gut microbiota, hepatic immune cells acquire the capacity to mount an effective immune response against HBV, leading to viral clearance [36]. This mechanism may explain the increased susceptibility of young children to persistent HBV infection compared to adults.

Recently, a large number of studies have begun to investigate the relationship between the role of intestinal flora and HBV infection and its progression. Chou et al. found that the gut microbiota contributes to HBV immunity and determines the outcome of HBV infection. Treatment with antibiotics impaired HBV-specific T cell response and resulted in persistent HBV infection in adult C3H/HeN mice [33]. Moreover, increasingly more evidence shows that the commensal microbiota participates in regulating the liver immune response. By using the hydrodynamic HBV transfection mouse model, researchers found that commensal microbiota affects the function of CD4+ T cells and maintains their anti-HBV infection effect [37]. Probiotics and their derived spermidine promote HBV clearance via autophagy-enhanced IFN-γ+CD4+ T cell immunity [38]. Overall, gut microbiota plays a critical role in the regulation of host immunity for HBV clearance.

## 4. Gut Microbiota Associated with HBV-Related Liver Disease

Interactions between hepatocyte hepatitis virus infection and the gut microbiome are bidirectional. While the gut microbiome can shape the immune responses against HBV infection, HBV infection can change the composition of the gut microbiome. Increasingly more scholars have recently begun to study the relationship between human intestinal flora and chronic HBV infection, liver cirrhosis, and HCC. Convincing evidence shows that changes in intestinal flora are related to the occurrence and development of liver disease (Table 1).

### 4.1. Chronic HBV Infection

A number of studies have proven that gut bacteria influence the progression of HBV-related disease not only through recognition of the pathogen-related molecular patterns (PAMPs), but also by sensing microbial metabolites. Spermidine derived from probiotics effectively promotes HBV clearance, which is likely to associate with more effective IFN-γ+CD4+ Th1 cells [38].Bacteria that produce butyric acid can help HBeAg-negative patients with chronic hepatitis B achieve functional cure by inhibiting the generation of hepatitis B virus through the effect of butyric acid [47]. In addition to the beneficial effects, we should pay special attention to the harmful effects of bacterial-derived PAMPs in CHB patients. Various studies have shown that not only can the cellular immune response caused by HBV invasion cause hepatocyte damage in chronic HBV infection, but also the natural immune response caused by PAMPs produced by intestinal microorganisms with microbial structure disorder [48,49]. One of the intestinal PAMPs associated with CHB is lipopolysaccharide (LPS), which is an endotoxin from the outer cell membrane of Gram-negative bacteria and is overproduced in acute or CHB patients [50,51]. Indeed, it is well established that there is an imbalance in the gut microbiota of CHB patients. For example, Xing et al. [52] found that the number of beneficial bacteria such as *Bifidobacterium* and *Lactobacillus* in CHB significantly reduced, while the levels of harmful bacteria such as *Enterobacteriaceae* and *Enterococcus* significantly increased. Notably, the researchers further found that the rapid decline in *bifidobacteria* and lactic acid bacteria in CHB patients was accompanied by a significant drop in the level of abundant unmethylated CpG DNA, which led to the weakening of the CpG DNA-TLR9 pathway, the reduction in the production of protective cytokines (especially IFN), and the fading of an immune effect against HBV infection [49,53,54]. In short, the disturbance of intestinal flora clearly affects the progress of CHB. Therefore, it is important to maintain the homeostasis of the intestinal microbiota of CHB patients.

### 4.2. Liver Cirrhosis

Alterations in the composition and abundance of intestinal flora can transfer pathological bacteria and their harmful products (such as LPS) directly to the liver through the gut–liver axis, which can activate the liver’s innate immune system, thereby affecting the transformation process of CHB to liver fibrosis [55]. Mechanically, intestinal permeability is further increased due to the destruction of tight junctions, and the medium that restricts the contact of bacteria with intestinal microvilli is reduced [55]. Abnormal bile acid levels are associated with hepatic fibrosis progression. Bile acids can promote hepatic stellate cell activation [56], as well as directly alter the gut microbiota by disrupting bacterial membranes and modulating lipid structures [57]. It is also known that sIgA deficiency can increase the permeability of the intestinal barrier [58,59]. In clinical practice, researchers found that the levels of sIgA and TNF-α in the fecal matter of patients with liver cirrhosis were significantly higher than those of CHB, asymptomatic HBV carriers, and healthy controls [53]. Mou H et al. [60] found that the level of serum IL-17A in patients with liver cirrhosis increased, and it participated in the process of liver inflammation in cooperation with *Enterococcus*, which revealed that IL-17A is widely involved in the pathogenesis and progression of chronic liver disease. In addition, a recent study showed that loss of *Akkermansia muciniphila* correlates with the abundance of hepatic monocytic myeloid-derived suppressor cells, and its reintroduction restores intestinal barrier function and strongly reduces liver inflammation and fibrosis [16]. Taken together, the deteriorating intestinal alterations could allow microbial metabolites and harmful antigen components to reach the liver, which would damage the immune response, accelerate intestinal dyskinesia, and exacerbate inflammation and liver fibrosis.

### 4.3. Hepatocellular Carcinoma

The change in the intestinal microbiota indicates the link between the effects of HBV, the immune response, and carcinogenesis in cirrhotic patients. The main role of gut microbiota seems to be to promote the development of HCC, rather than its induction. In fact, gut dysbiosis is a negative prognostic factor related to the risk of HBV infection or non-infection of HCC [61]. In CHB patients, it is generally believed that HBV infection initiates the entire pathophysiological cascade resulting in the development of HCC. In addition, the immune activation related to HBV infection causes impaired intestinal permeability and changes the intestinal microbiota composition that perpetuates this chronic inflammatory condition through the activation of TLR (namely TLR4 activation) [62]. Indeed, TLR4 is present in multiple liver-resident cells (including hepatocytes, HSCs, and Kupffer cells), which is activated by the translocation of bacterial components called PAMPs, responsible for the promotion of hepatocarcinogenesis [63]. Thus, the alterations of gut microbiota are strongly linked with the progression of HCC. In fact, some alterations of the intestinal microbiota in HBV infection patients are specific. For example, patients in the end stage of CHB show a higher growth of E. coli and increased levels of LPS ligand in circulation [53]. Consistently, recent data also demonstrated that SCFA-producing bacteria were significantly diminished, whereas LPS-producing genera were enhanced in patients with HCC compared with controls [64]. Notably, a study showed that probiotic administration can significantly decrease LPS and reduce the size, number and development risk of HCC in a rat model of liver cirrhosis [65]. Furthermore, intestinal barrier impairment and bacterial translocation are defined as key mechanisms that shape the hepatic inflammatory microenvironment and fuel liver disease progression towards cirrhosis and HCC [13]. Quite evidently, the capacity of the microbiota and its metabolites to modulate immune responses has been linked to improved prognostic outcomes in HCC [66]. Therefore, it appears that gut dysbiosis may be a possible future maker for the estimation of HCC development risk, and reverse intestinal dysbiosis is a promising target for the treatment and prevention of HBV-related liver cirrhosis and HCC.

**Figure 2:**
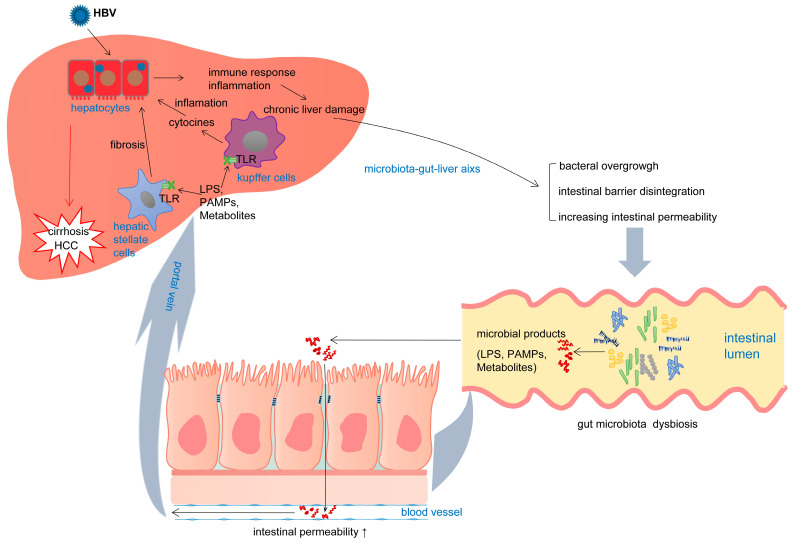
Effects of the gut microbiota on HBV-related liver disease. Chronic HBV infection results in microbiota dysbiosis and intestinal bacterial overgrowth via the gut–liver axis. Alterations in the intestinal microbiota can cause intestinal barrier disintegration and increase intestinal permeability. Meanwhile, disordered gut microbiota releases a large number of toxic metabolites, including LPS, PAMPs, and deoxycholic acid. LPSs then activate TLRs, which are expressed on Kupffer and stellate cells. Toxic metabolites transfer from the gut to the liver and combine with HSC in the liver, leading to the transformation of HSC into a senescence-associated secretory phenotype. Activation of these cells, associated with HBV damage, stimulates an immune response with proinflammatory cytokine production and liver fibrosis generation, which eventually lead to the occurrence and progression of liver cirrhosis and HCC.

Taken together, the mechanism causing HBV to promote liver disease is partly mediated through intestinal microbiota. In CHB patients, changes in intestinal permeability accompanied by bacterial translocation and endotoxin entry into the portal vein led to increased intrahepatic-related signaling pathways in the liver, increasing production of consequential cytokine and inflammatory factors, causing liver damage, and ultimately exacerbating the development of fibrosis, liver cirrhosis, and liver cancer (Figure 2).

## 5. Gut Microbiota and Pharmacotherapy

Nucleoside (acid) analogs, including entecavir (ETV), tenofovir disoproxil fumarate, and tenofovir alafenamide, are recommended for CHB treatment. Microbiota have been demonstrated to be among the vital factors governing HBV-related liver disease progression and response to therapy in patients. Gut microbiota composition was altered in a persistent hepatitis B virus infection mouse model. Entecavir therapy could restore HBV infection-induced gut microbiota dysbiosis [67]. This indicates that the regulation of the intestinal microbiota by ETV helps restore the intestinal microbiota, thereby influencing therapeutic outcome. Subsequent clinical studies revealed that there were specific changes in bacterial species during the treatment with ETV. Depletion of the *E. hallii* group and *Blautia* was confirmed in treatment-naïve patients, whilst it was restored in patients with ETV treatment compared to healthy controls [68]. In addition to the significant impact of entecavir on the intestinal microbiota of CHB patients, researchers found that TAF also had an impact on the intestinal microbiota of CHB patients [69]. These findings provide new insights into the role of gut microbiota in the treatment of CHB patients with oral antiviral drugs.

## 6. Gut Microbiota Modulation in CHB Therapy

Alterations in gut microbiota seem to play an important role in the induction and promotion of HBV-related liver disease. It stands to reason, then, that intestinal dysbiosis that could impact on the balance of microbes in the gut could be impactful on the course of CHB. Targeting gut dysbiosis for remission induction, maintenance, and recurrence prevention is an attractive treatment for HBV-related liver disease. Intervention strategies targeting the gut microbiota and their subsequent translation into clinical trials are already underway.

### 6.1. Targeted Therapy with Probiotics

A growing body of clinical evidence suggests that intestinal microbiome regulation can improve the status of liver diseases, especially related to liver cirrhosis and its complications (such as hepatic encephalopathy, spontaneous bacterial peritonitis, and other infections). Recently, in the context of the accumulating benefits of regulating gut microbiota through the use of probiotics, treatment strategies targeting gut microbiota for chronic liver disease have received a high level of attention [70,71]. The rationale for using probiotics in this pathogenic environment comes from their beneficial gut microbiota regulation, immune stimulation, and regulation, as well as their ability to reduce the intestinal permeability of LPS and other PAMPs [72,73]. Currently, although consistent results have been found in experimental studies and clinical practice on the beneficial effects of probiotics in multiple liver diseases, there are few clinical data on CHB and HCC. Probiotics have been proven to reduce serum HBsAg in patients with low HBsAg levels [38]. Furthermore, Xiaoxue Xia et al. [74] investigated the effect of probiotics (*Clostridium butyricum* combined with *Bifidobacterium* infantis) on minimal hepatic encephalopathy in patients with HBV-induced liver cirrhosis. They observed that the dominant bacteria (*Clostridium cluster I* and *Bifidobacterium*) of the probiotics-treated group were significantly enriched, while *Enterococcus* and *Enterobacteriaceae* were significantly reduced. Importantly, the probiotic treatment significantly reduced venous ammonia, ameliorated the intestinal mucosal barrier, and improved patients’ cognition. A deeper understanding of the mechanism of action of the intestinal microbiota in the formation of complications of HBV-related liver diseases has made probiotic supplements an attractive treatment strategy for such patients. Patients with liver cirrhosis are considered immunocompromised and therefore may be more vulnerable to the actual or theoretical risks associated with ingesting living bacteria [75]. Although current studies have shown that the use of probiotics in patients with HBV infection is safe, caution is still necessary given the reports of sepsis in critically ill patients [76] and the increased risk of infection in patients with advanced liver disease.

### 6.2. Fecal Microbiota Transplantation

FMT has now become a highly promising therapeutic approach, with the potential to restore the composition and metabolic functions of the intestinal microbiota [77,78,79,80]. More emerging evidence indicates that the application of FMT can improve the progression of HBV-related chronic liver disease. For example, Yan-Dan Ren et al. carried out a trial of FMT for the treatment of HBeAg-positive CHB in patients with ongoing Tenofovir/ETV therapy. They demonstrated that FMT can induce HBeAg clearance in a significant proportion of the cases that have persistent positive HBeAg even after long-term antiviral treatment [81]. This study provides evidence that regulating gut microbiota is beneficial for CHB treatment, but larger trials are needed in the future. Although there are few studies on the use of FMT in the treatment of CHB and its related complications, we know that cirrhotic patients induced by different causes are usually complicated by hepatic encephalopathy (HE), spontaneous bacterial peritonitis, and other infections due to microbiota dysbiosis and increased gut permeability. An open-label, randomized clinical trial in patients with cirrhosis with recurrent HE was conducted by Bajaj JS’s groups, who found that the rational selection of donor FMT can reduce the number of hospitalizations in patients with liver cirrhosis and recurrent HE, and improve cognition and ecological dysfunction [70]. FMT has not yet been clinically applied in patients with HCC, but promising results have been achieved in studies of other cancers (such as melanoma and colorectal cancer) [82,83]. In particular, studies on pancreatic cancer have shown that regulating the intestinal microbial community through FMT can enhance the efficacy of immunotherapies such as immune checkpoint inhibitors and restore anti-tumor immunity in animal models and early clinical trials [84,85]. These findings suggest that FMT may play a role as an adjunctive therapy for HCC by reshaping the intestinal microbial community to support immune function. Moreover, FMT has not been observed to increase the incidence of adverse events in liver diseases [86,87]. Thus, FMT may be a potentially useful therapy for HBV-related disease and rigorous clinical studies are needed to evaluate its safety, feasibility, and therapeutic effect in future.

## 7. Conclusions and Perspective

Briefly, this review of the intestinal microbiota indicates that the adverse changes in microbiota—that is, microbiota dysbiosis—is closely correlated with the pathogenesis and progression of chronic HBV infection. In particular, changes in the gut microbiota can potentially drive the progression of CHB to severe forms of liver damage until the development of liver cirrhosis and HCC. The importance of intestinal microbiota in HBV infection and HBV-related liver disease not only expands our understanding of the indispensable role of microbiota in liver disease from a novel perspective, but might also help change the treatment strategy for CHB patients with intestinal dysbiosis. In the future, new discoveries in the field of the intestinal microbiota will certainly contribute to the development of innovative therapeutic approaches for HBV-related liver disease. In addition, careful prospective studies are required to provide evidence for a causal relationship between microbial species in CHB development.

## Figures and Tables

**Table 1:** Microbiota dysbiosis in CHB and outcomes of dysbiosis.

Microbiota Dysbiosis	Outcomes	References
*Veillonellaceae↑* *Lachnospiraceae↑* *Rikenellaceae↑* *Porphyromonadaceae*↓ *Ruminococcaceae*↓	Detractive intestinal barrier against the colonization of pathogenic microbes by reducing the production of SCFAs and antimicrobial peptides	[39,40]
*Enterobacteriaceae↑* *Veillonella↑* *Bacteroidetes*↓	Correlated with the Child-Turcotte-Pugh scores	[41]
*Prevotella*↑ *Alloprevotella*↑ *Faecalibacterium↑* *Ruminiclostridium↑*	Increased anti-inflammatory bacteria may be in response to HBV infection.	[42]
The detection rate of intestinal fungi and the diversity of enteric fungi increased	Positively correlated with the disease progression of patients with different degrees of chronic HBV infection	[43]
*Bifidobacterium* dysbiosis *(B.dentium↑* *B.catenulatum group*↓ *B. longum↓)*	Intestinal Bifidobacterium species might shift from beneficial ones to opportunistic pathogens that associated with CHB and HBV-related cirrhosis	[44]
The diversity of *lactobacilli* population↓	Disturbance of lactobacilli flora is associated with the liver injury	[45]
Increased the ratio of Bacteroidetes to Firmicutes *(Firmicutes*↓ and *Bacteroidetes↑)*	Associated with inflammatory disorders and may accelerate HBV-induced chronic liver disease progression	[46]

HBV, Hepatitis B virus; CHB, chronic hepatitis B; SAFCs short-chain fatty acids; “↑”, represents the increased abundance of the corresponding bacteria; “↓”, represents the decreased abundance of the corresponding bacteria.

## Data Availability

No new data were created or analyzed in this study. Data sharing is not applicable to this article.
